# Experimental Study and Numerical Simulation on the Influence of Specimen Size on Failure Characteristics and Mechanics of Plastic Concrete Under the Uniaxial Compression Test

**DOI:** 10.3390/ma17235986

**Published:** 2024-12-06

**Authors:** Xuwei Pan, Mingjian Guo, Siwei Wang, Tong Jiang, Shuai Liu, Shuo Zhang

**Affiliations:** College of Geosciences and Engineering, North China University of Water Resources and Electric Power, Zhengzhou 450046, China; panxuwei@ncwu.edu.cn (X.P.); 18579884946@163.com (M.G.); wangsiwei@ncwu.edu.cn (S.W.); jiangtong@ncwu.edu.cn (T.J.); liushuai@ncwu.edu.cn (S.L.)

**Keywords:** plastic concrete, uniaxial compression test, numerical simulation, size effect, failure characteristics and mechanics

## Abstract

In this paper, uniaxial compression tests and numerical simulation were conducted on specimens of five sizes, and the influence of specimen size on the failure characteristics and mechanics of specimens was studied. The results show that when the bottom size of the specimen is the same, with the increase in the height–width ratio of the specimen size (from 1 to 3), the peak stress of the specimen gradually decreases, but when the decrease is greatly reduced, the concentration of contact force chains in the model increases. The failure mode of the specimen changes from tensile failure to shear failure, and the distribution of cracks changes from multiple vertical cracks uniformly to a concentrated main oblique crack. The failure characteristics change from the overall failure to the serious failure of the near stressed end of the specimen, while the far stressed end is not failure or slight failure. When the height–width ratio of the specimen is the same, with the increase in the overall size, the peak stress decreases, and the dense vertical cracks change into a small amount of concentrated oblique cracks, and the integrity of the specimen and model is better. There is a good effect using PFC2D software to simulate the crack evolution and failure characteristics of plastic concrete.

## 1. Introduction

Plastic concrete is a flexible engineering material composed of bentonite, cement, sand, gravel, and water, which is mixed with a high water–cement ratio. Its main characteristics are as follows: low elastic modulus, which is similar to the elastic modulus of the foundation; it can better adapt to the deformation of foundation, allowing the plastic concrete structure to bear combined forces with the foundation; and good crack resistance performance. The permeability coefficient is small, which reduces the water seepage of the structure. The mixture has good workability, self-leveling, and self-compacting, without vibration. The early strength is low, and the later strength increases rapidly. As an anti-seepage engineering material, plastic concrete is widely used in earth–rock dams, reservoirs, cofferdams, and other projects [1,2,3,4,5,6,7,8,9]. Less cement consumption can save a large amount of cement and reduce the project cost and makes materials more in line with low-carbon requirements.

Size effect refers to the phenomenon that the mechanical properties of rock materials change with the change in sample size, which has a significant impact on the mechanical behavior of rock, thus affecting the safety and stability of rock engineering. It is of great significance to study the size effect of rock mass for rock engineering.

Scholars have carried out a lot of experimental and theoretical research on the characteristics of plastic concrete. The research mainly focuses on the preparation method and mix ratio [10,11,12,13,14] of plastic concrete, mechanical characteristics [15,16,17,18,19,20,21,22], failure deformation characteristics, and impermeability [23,24,25,26,27,28]. Song conducted true triaxial compression tests on plastic concrete, studied failure modes and constitutive curves, and proposed failure criteria for plastic concrete under true triaxial compression [1]. Many scholars have carried out a lot of work on the study of size effect starting with eighties of the last century within the Fracture Mechanics Theories developed for many engineering materials [29,30,31,32,33]. Zhou [34] conducted static load tests on rock specimens with different aspect ratios using the RMT rock mechanics testing system. Studies have shown that the larger the rock size, the more broken the fragments. Zhai [35] studied the size effect of two different types. Sinha [36] studied the size effect of limestone by conducting uniaxial compression tests on cube specimens of different sizes, and pointed out that porosity would affect the size effect. Hu [37,38,39,40] studied the influence of joint number on the size effect of modulus, the influence of rough joint rock size on uniaxial compressive strength, the influence of roughness size effect on UCS, and the influence of joint number and size on strength. Zhao [41] studied the size effect of bulk modulus and pointed out that there is a negative correlation between modulus and size. Ankah [42] studied the influence of roughness heterogeneity on joint size effects and pointed out that rock joint heterogeneity controls scale effects. Han’s [43] research suggested that the size effect of failure mode under true triaxial compression is weak, and the size effect of mechanical properties is significant. Different lithology has an effect on the size effect. The size effect of coal rock was studied from strength, deformation, and acoustic emission characteristics [44]. Choo [45] studied the effect of size on the mechanical properties and crack propagation of rock-containing cracks. The results show that the size has a great influence on the uniaxial compressive strength and cracking mode. The study showed that the size effect appeared in most experiments, especially in strength. A formula for the size effect of concrete is established in [46].

More and more new technologies and methods are being applied to study size effects. Ferretti [47] used theoretical analysis, experiments, numerical simulations, and other methods to study the shape effect in the effective laws of plain concrete and rubber concrete. Wang [48] used 3D printing technology to study the size effect of rock. The research shows that the size has a significant effect on the mechanical properties and failure modes. Three-dimensional printing technology is used to analyze the effect of size effect on the strength and failure of rock-containing cracks [49]. Yang [50] used a numerical simulation method to study the size effect of permeability and mechanical properties of rock mass containing joints. Studies have shown that the observed scale has a significant effect on the permeability of rock mass, and the mechanical properties are more sensitive to size than permeability. The numerical calculation method and the statistical method are combined to analyze the size effect of the mechanical properties of the jointed rock mass, and the mathematical relationship between the specimen size and the elastic modulus and the joint is established [51]. Based on the analysis of numerical calculation method, Zhong pointed out that the mechanical parameters of rock mass have significant size effect [52]. Cai [53] studied the influence of specimen size on material permeability, and pointed out that permeability was negatively correlated with specimen size.

There is a large number of tests and studies on the size effect of concrete. Ince [54] tested three different depths (size range = 1:4) and two different height/depth ratios 2 and 3 under concentrated load. The results show that the size effect is very consistent with the modified size effect law (MSEL). The larger concrete specimens have a stronger strain rate effect, and the increase in strain rate will weaken the influence of specimen size on the actual compressive strength of concrete [55]. Yu’s [56] research shows that the compressive strength of lightweight concrete gradually decreases with the increase in cube size (70, 100, and 150 mm). Jin’s [57] research shows that the splitting tensile strength decreases with the increase in side length, and the decreasing trend of strength decreases with the increase in side length. The concrete specimens with a higher strength grade have a more obvious strength decline trend than those with a lower strength grade. Liu’s [58] research shows that the critical strain rate is 1/s, which weakens the influence of size effect. When the strain rate reaches 10/s, the dynamic compressive strength of recycled concrete increases with the increase in section size.

Some theories and models related to size effects have been proposed. A model of size effect is proposed for igneous rock, which is used to analyze the influence of specimen size on strength [59]. Qi [60,61] studied the influence of size effect and loading rate interaction on the mechanical properties of rock mass, and established the relationship between UCS, loading rate, and size. Based on the classical fracture mechanics theory, Zhao [62] proposed the rock failure criterion, studied the size effect of the internal cracks of the rock mass, and analyzed the influencing factors of the size effect. Li [63] deeply analyzed the influence mechanism of size effect from the microscopic point of view, and pointed out that the latter is not the mechanical parameters such as strength and cohesion of rock. Li [64] studied the size effect and failure mechanism of slate based on laboratory tests. The results show that the specimen size has a significant relationship with the modulus of isotropic slate. Based on the mathematical statistical method, the relationship between the specimen size and the deformation characteristics of jointed rock mass is studied [65]. Ma [66] studied the strength parameters and size effects of randomly jointed limestone rock masses. The research results indicate that the mechanical parameters of rock begin to fluctuate significantly with the increase in size. The size has an effect on the mechanical properties of concrete specimens, and the loading rate during the test will affect the size effect on the strength [67].

The main conclusion is that the strength decreases with the increase in the size. The existing research obviously cannot fully reveal the influence of specimen size on the failure characteristics and strength of plastic concrete under uniaxial compression. In this paper, uniaxial compression tests and numerical simulation were carried out on plastic specimens with five sizes to study the influence of specimen size on the failure characteristics and mechanics.

## 2. Experiment

### 2.1. Specimen Preparation

The cement used in this study is ordinary Portland cement with a grade of 42.5 produced by Longgang Cement Plant in Zhengzhou City, Henan Province, China. The main indicators of the cement as shown in Table 1. The bentonite used in this study is calcium-based bentonite from Xinyang City, Henan Province, China. The main indicators as shown in Table 2. The sand used in this study is natural river sand. The results of the particle screening test for sand as shown in Table 3. The gravel used in this study is crushed stone with a particle size of 5–20 mm. The results of the particle screening test for the crushed stones as shown in Table 4. The mix proportion design is carried out according to the assumed bulk density method, which is shown in Table 5. Slump and diffusivity are the evaluation indexes of workability and fluidity of plastic concrete mixture, respectively. The two tests of slump and diffusivity can be carried out simultaneously by slump cylinder. The slump and diffusivity of the plastic concrete mixture used in this study are 19.8 cm and 35.3 cm, respectively.

The mixer is cleaned before mixing, and the inner wall of the mixer is soaked with water. Firstly, the weighed stone and sand are put into the mixer for 1 min, and the sand and stone are mixed evenly. Secondly, the weighed cement and bentonite are put into the mixer and mixed for 1–2 min to mix them evenly. Finally, water is added in batches. Most of the water is first added, and the state of the mixture in the mixer is observed while stirring, and then the remaining water is added as appropriate, stirring for 2–3 min.

The specimens used in this study consist of cube specimens with side lengths of 100 mm and 150 mm, as well as prism specimens with dimensions of 100 mm × 100 mm × 200 mm, 100 mm × 100 mm × 300 mm, and 150 mm × 150 mm × 300 mm. The inner wall of the mold is oiled in advance. The mixture is then poured into the molds, and the molds are vibrated at the corners to ensure thorough filling of the mixture. The surface of the pour is subsequently smoothed and covered with plastic film to prevent water evaporation. After standing for a period of 24 h, the molds are carefully removed. It is important to handle the molds gently during the removal process to avoid any internal material damage or chipping of edges and corners. Finally, the specimens are placed in a standard curing room with a temperature of 20 ± 3 °C and humidity of ≥ 90% for a duration of 28 days.

### 2.2. Uniaxial Compression Test

**Figure 1:**
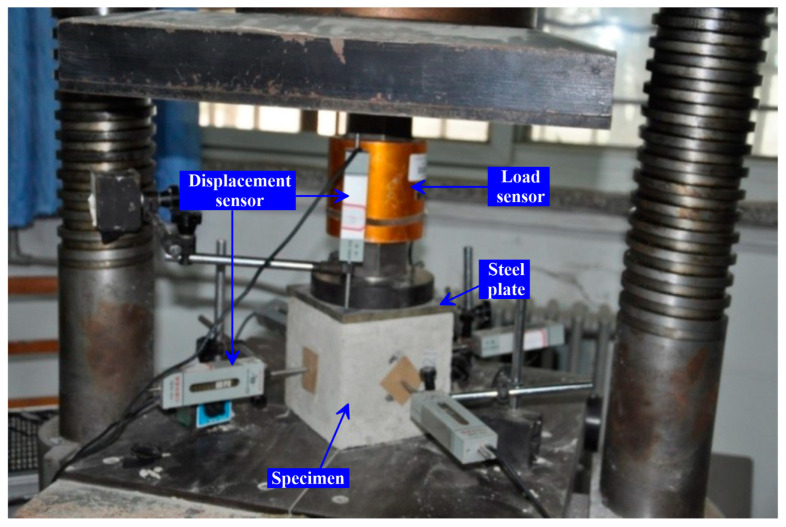
Uniaxial compression test.

A pressure testing machine (Hebei Zhongke Beigong Test Instrument Co., Ltd., Cangzhou, China) with a range of 200 kN is used in the uniaxial compression test. The specimen of 28 days age was placed in the middle of the upper and lower pressure plates of the testing machine, and the steel plate was placed on the top surface of the specimen. The axial displacement of the specimen is measured by an electronic displacement meter installed on the upper part of the steel plate, and the lateral displacement is measured by four electronic displacement meters (erected perpendicular to the four sides of the specimen). The load sensor is placed between the steel plate and the upper pressure plate of the testing machine to measure the load data. The load and displacement data are imported into the computer. After the test instruments are correctly installed in place, the test machine is started, and the loading rate is controlled to be 0.01 mm/min until the specimen is failure. The uniaxial compression test is shown in Figure 1.

## 3. Test Results and Analysis

### 3.1. Influence of Specimen Size on Failure Characteristics

**Figure 2:**
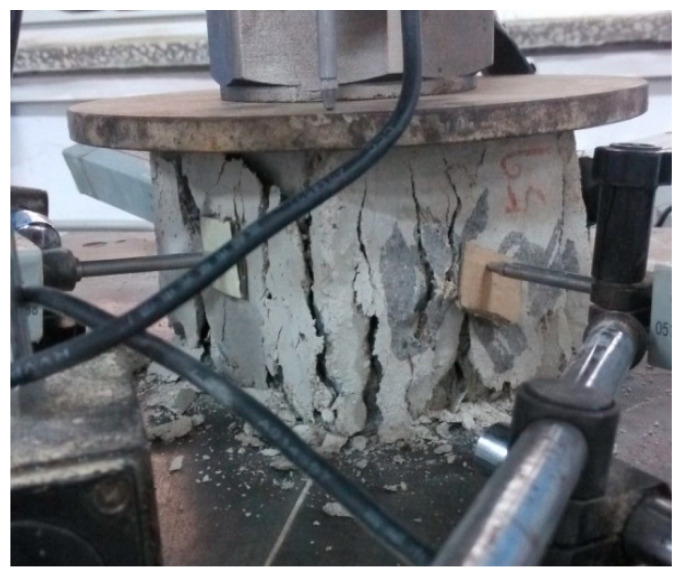
The specimen with a size of 100 mm × 100 mm × 100 mm.

The failure of the specimen with a size of 100 mm × 100 mm × 100 mm and a height-width ratio of 1 is shown in Figure 2. The vertical cracks on the outer surface of the specimen are dense, the crack width is large, the distribution is uniform, and the direction is parallel to the compression direction. The cracks penetrate the entire outer surface of the specimen. The specimen is mainly tensile failure, and the shear failure is not obvious. The overall failure is very serious and thorough. The outer surface of the specimen is almost all upturned and detached, and the coarse aggregate is separated from the cementitious material.

**Figure 3:**
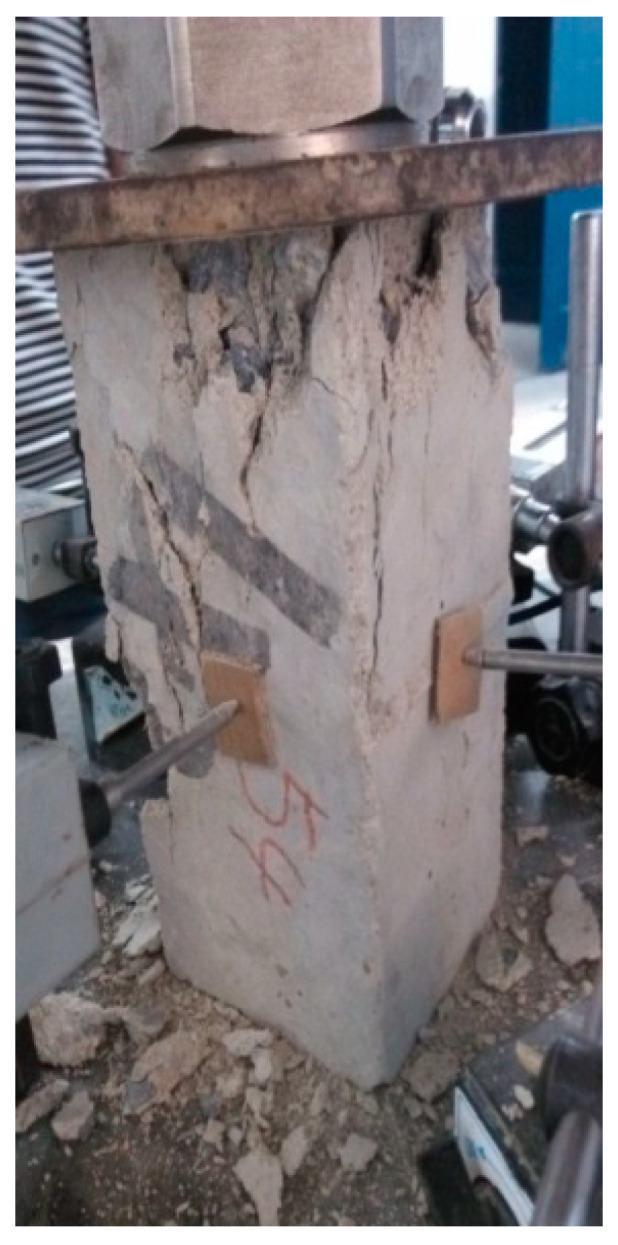
The specimen with a size of 100 mm × 100 mm × 200 mm.

The failure of the specimen with the size of 100 mm × 100 mm × 200 mm and the height-width ratio of 2 is shown in Figure 3. There is an obvious oblique crack on the outer surface of the specimen, which penetrates diagonally from the top angle of the specimen, and the shear failure characteristics are obvious. The upper part of the specimen is seriously failure, and the coarse aggregate is separated from the cementitious material and exposed. The bottom of the specimen is intact and not a failure. The overall integrity of the specimen after failure is more complete than that of the specimen with a size of 100 mm × 100 mm × 100 mm. Because the cylinder of the pressure testing machine is located at the bottom, that is, the part where the specimen is a serious failure is the near stressed end, and the integrity of the far stressed end is better.

**Figure 4:**
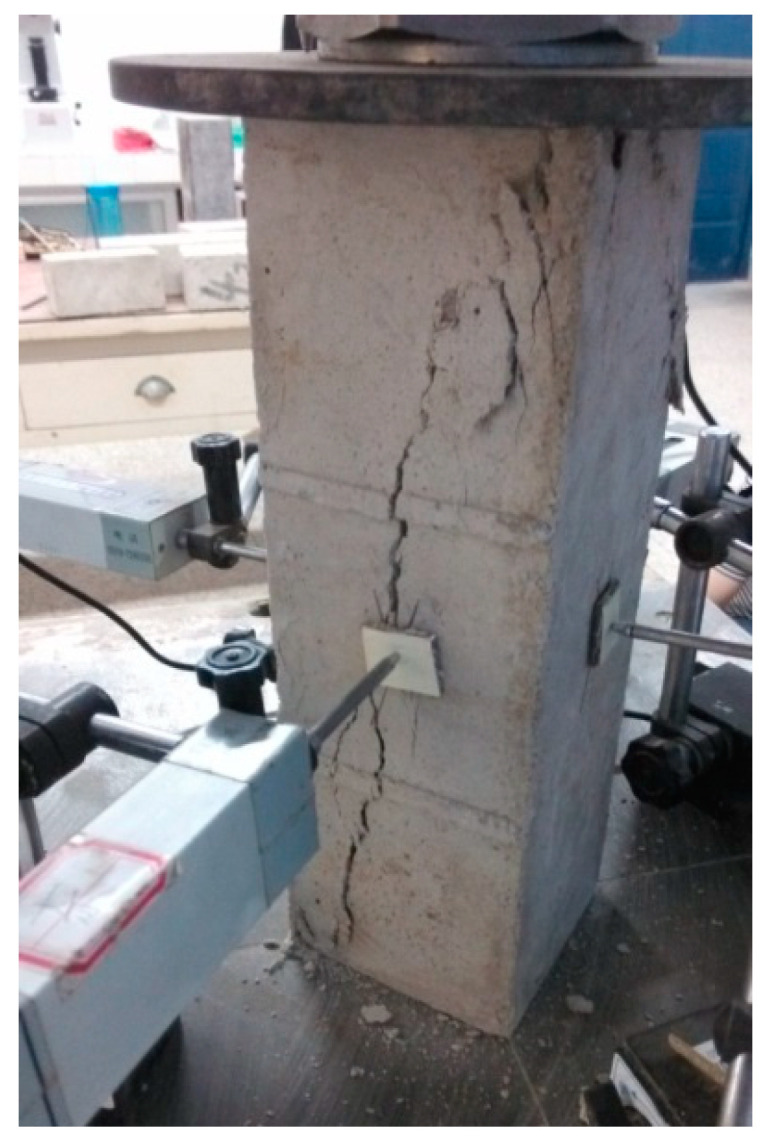
The specimen with a size of 100 mm × 100 mm × 300 mm.

The failure of the specimen with the size of 100 mm × 100 mm × 300 mm and the height–width ratio of 3 is shown in Figure 4. There is an obvious oblique crack on the outer surface of the specimen, which penetrates from the top to the bottom of the specimen, and the shear failure characteristics are obvious. The upper part of the specimen is an obvious and serious failure, but only a small number of ups fall off, and the coarse aggregate is not exposed. The bottom of the specimen is complete and is not a failure. The overall integrity of the specimen after failure is more complete than that of the specimen with a size of 100 mm × 100 mm × 200 mm.

**Figure 5:**
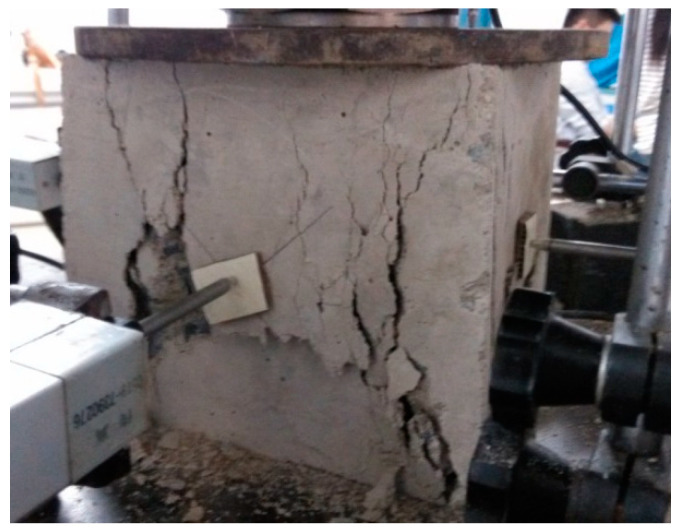
The specimen with a size of 150 mm × 150 mm × 150 mm.

The failure of the specimen with the size of 150 mm × 150 mm × 150 mm and the height–width ratio of 1 is shown in Figure 5. The oblique cracks on the surface of the specimen are formed by the oblique expansion and penetration of multiple vertical cracks. The width and depth of the crack are large, extending from the four top angles to the center of the specimen. The surface of the specimen is seriously peeled, and the coarse aggregate at the crack is exposed and separated from the cementitious material. The overall failure of the specimen is more serious, and the overall integrity is more complete than the specimen with a size of 100 mm × 100 mm × 100 mm.

**Figure 6:**
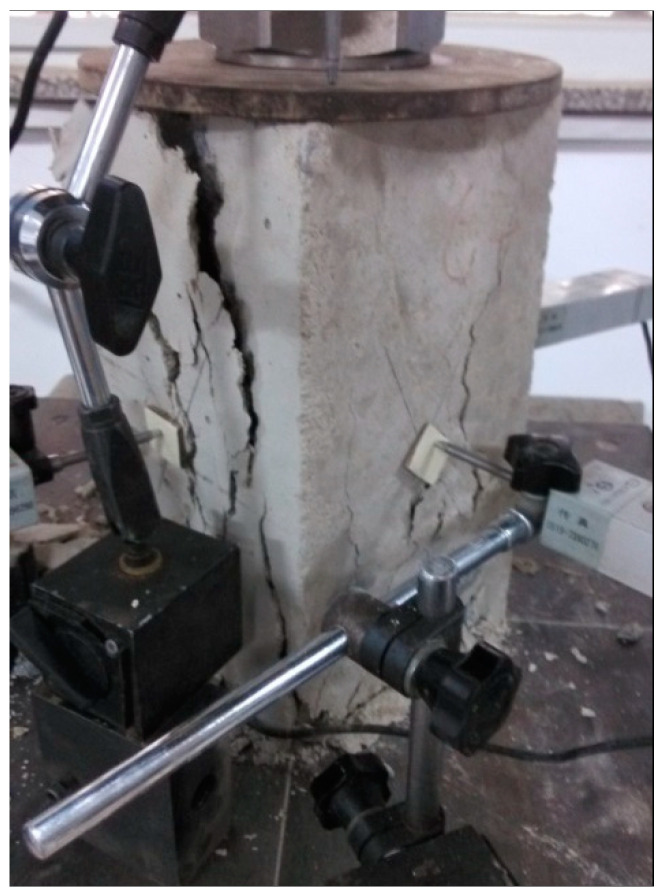
The specimen with a size of 150 mm × 150 mm × 300 mm.

The failure of the specimen with the size of 150 mm × 150 mm × 300 mm and the height–width ratio of 2 is shown in Figure 6. There is a wide oblique crack on the surface of the specimen. The crack penetrates from the top of the specimen to the bottom. The widest part of the crack is about 3 cm, and the deepest part can be up to 3–4 cm. The two oblique cracks in the adjacent surface have been penetrated, and the failure of the specimen is extremely serious. Large amounts of coarse aggregate fall off. The top of the specimen shows the most severe failure, the bottom is relatively complete, and the shear failure characteristics are obvious. The overall integrity of the specimen after failure is more complete than that of the specimen with a size of 100 mm × 100 mm × 200 mm.

### 3.2. Influence of Specimen Size on Mechanics

**Figure 7:**
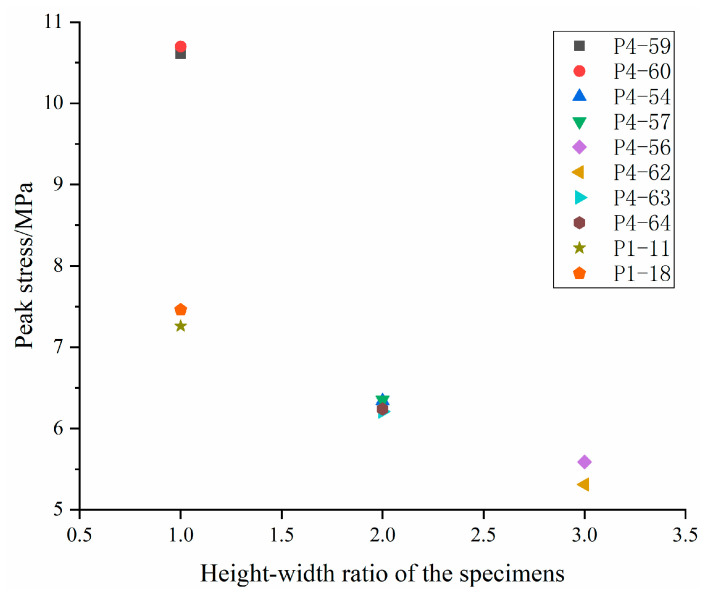
Peak stress of the specimens.

The peak stress of the specimens with five sizes under uniaxial compression is shown in Table 6 and Figure 7. When the bottom width of the specimen is 100 mm and the height is 100 mm, 200 mm, and 300 mm, the average peak stress is 10.66 MPa, 6.34 MPa, and 4.45 MPa, respectively. When the bottom width of the specimen is same, with the increase in the height–width ratio, the peak stress of the specimen decreases gradually, with a decrease of 40.5% and 29.8%, respectively, and the decrease is greatly reduced. When the height–width ratio is the same, the peak stress of the specimen decreases with the increase in the overall size. When the height–width ratio of the specimen is 1, the peak stress of the cube specimen with a side length of 100 mm, 150 mm is 10.66 MPa, and 7.36 MPa, respectively, and the strength is reduced by 30.9%.

### 3.3. The Axial Stress–Strain Curve

**Figure 8:**
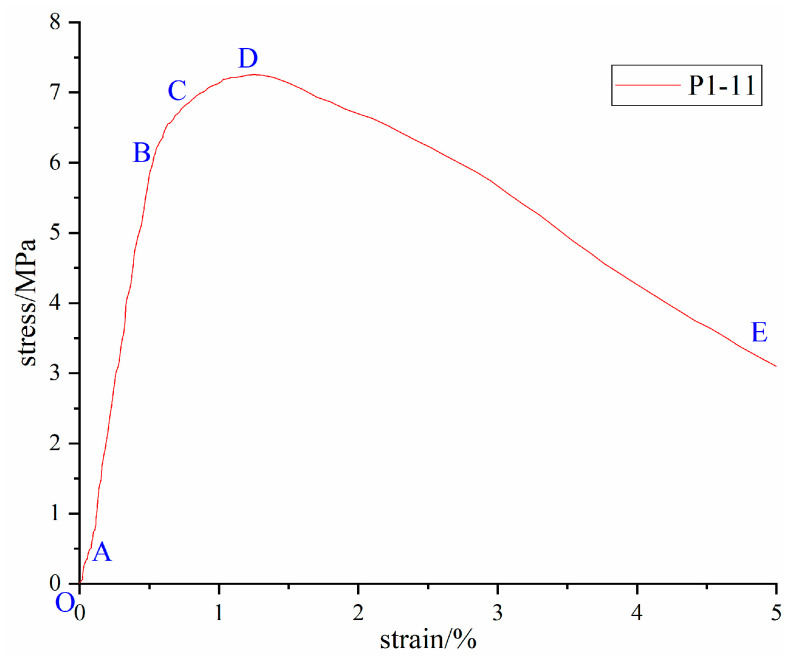
The axial stress–strain curve of specimen P1-11.

The axial stress–strain curve of specimen P1-11 is shown in Figure 8, and the curve includes five stages. In the initial loading section (OA section), the specimen is compacted, and the volume is compressed. In the straight rising section (AB section), the slope of the AB section is the elastic modulus, and point B is called the proportional limit point. In the stable fracture section (BC section), the increase rate of axial strain decreases with the increase in load increases, and the volume compression rate decreases with the increase in load. Point C corresponds to the inflection point of volume strain–axial strain curve. At point C, the volume is compressed to the minimum, and the volume begins to change from compression deformation to expansion deformation. Point C is the volume expansion point and the boundary point between the stable rupture section and the unstable rupture section. The CD section is the unstable rupture stage, point D is the peak point. The DE section is the descending section; the stress decreases with the increases in strain. The specimen size has no significant influence on the stress–strain curve.

## 4. Numerical Simulation

### 4.1. Determination of Model Parameters

**Figure 9:**
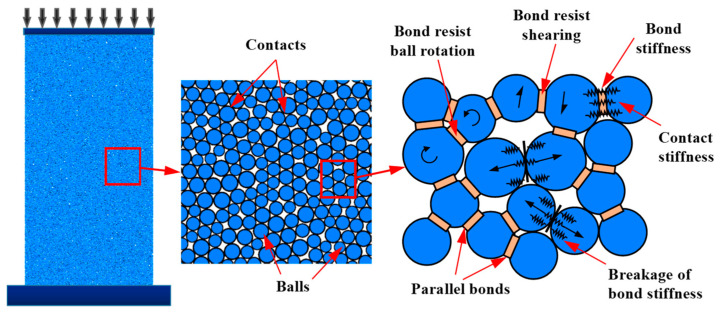
The typical uniaxial compression numerical model and internal parallel bonding contact diagram in the PFC2D program.

**Figure 10:**
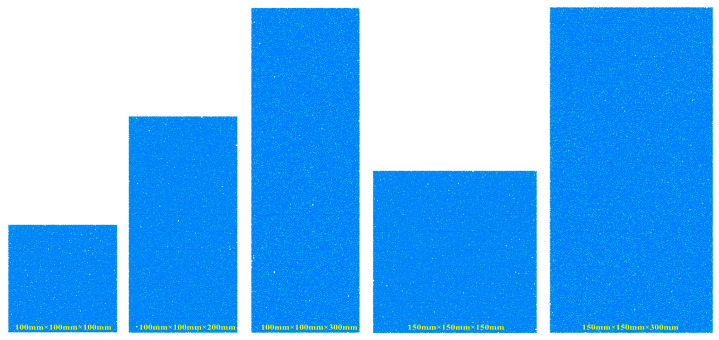
Numerical simulation model.

Compared to macroscopic laboratory experiments, PFC2D software (version 5.0) is suitable for analyzing the microscopic characteristics of the progressive rock failure process, such as crack evolution, stress concentration areas, etc. It is currently a very reliable numerical simulation method in the field of rock mechanics [68,69,70]. The basic unit particles used in PFC2D are balls, and the interactions between particles are connected using a contact model. The parallel bonding model can simultaneously transmit force and torque, and has a large contact range, making it suitable for simulating rock materials [71,72]. The typical uniaxial compression numerical model and internal parallel bonding contact diagram in the PFC2D program are shown in Figure 9. The numerical simulation model is established by using the parallel bonding model [73,74,75], as shown in Figure 10.

Before establishing a numerical model for simulation, it is necessary to conduct numerical simulation experiments by constructing the same model as the indoor experiment and comparing it to ensure consistency between the indoor experiment and numerical simulation results [76,77].

Using the same testing method and loading conditions as indoor experiments, the stress–strain curve obtained in the PFC2D program was verified, and the microscopic parameters suitable for PFC2D simulation were obtained, as shown in Table 7.

**Figure 11:**
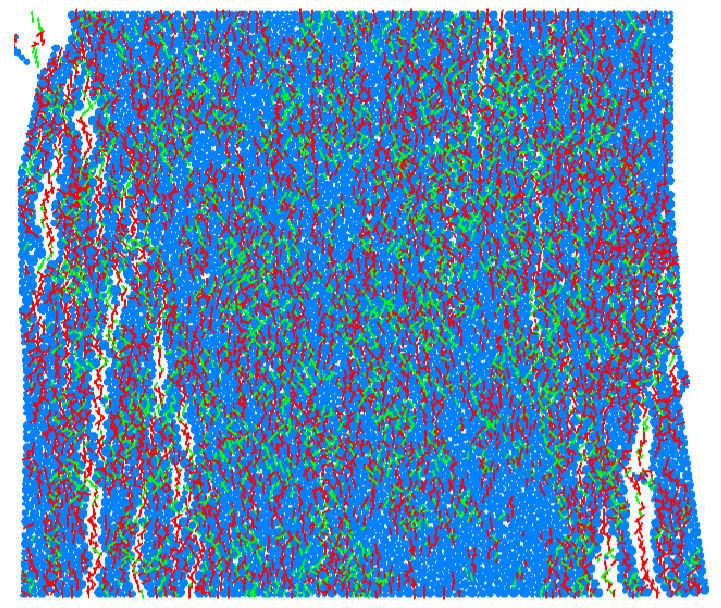
Failure mode of the specimen with size of 100 mm × 100 mm × 100 mm.

**Figure 12:**
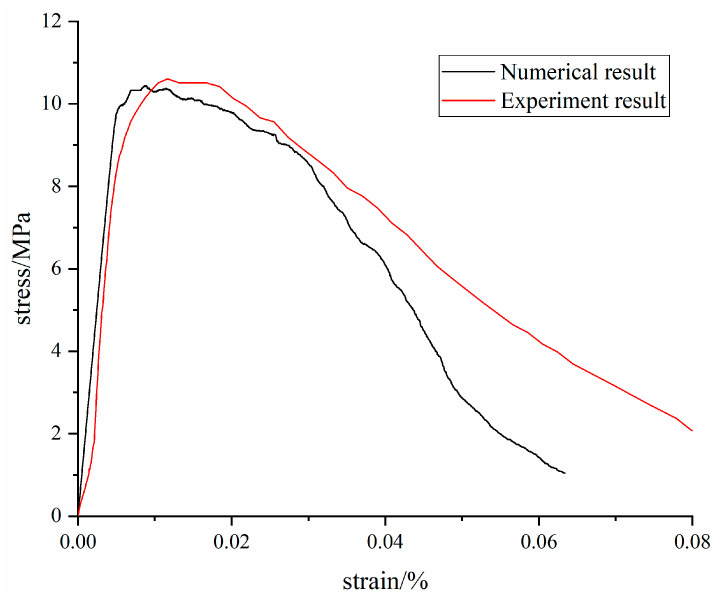
Verification of experimental and numerical results.

Taking the specimen with a size of 100 mm × 100 mm × 100 mm as an example, the final failure mode of the specimen obtained by numerical simulation is shown in Figure 11, where red represents tensile cracks and green represents shear cracks. Vertical cracks are dense and are present throughout the model. When the specimen is a failure (i.e., after peak stress), tensioning cracks are predominant, with a small number of shear cracks. The overall damage to the model is serious, and some spheres have separated from the model. The numerical simulation is basically consistent with the experiment (Figure 11). The stress–strain curve obtained from numerical simulation is shown in Figure 12, and its peak strength and deformation modulus are basically consistent with the experiment. The curve shape is in good agreement with the experimental curve. It shows that the model parameters used in this numerical simulation are reasonable and can be used to simulate this experiment.

### 4.2. Analysis of Crack Evolution and Failure Mode

**Figure 13:**
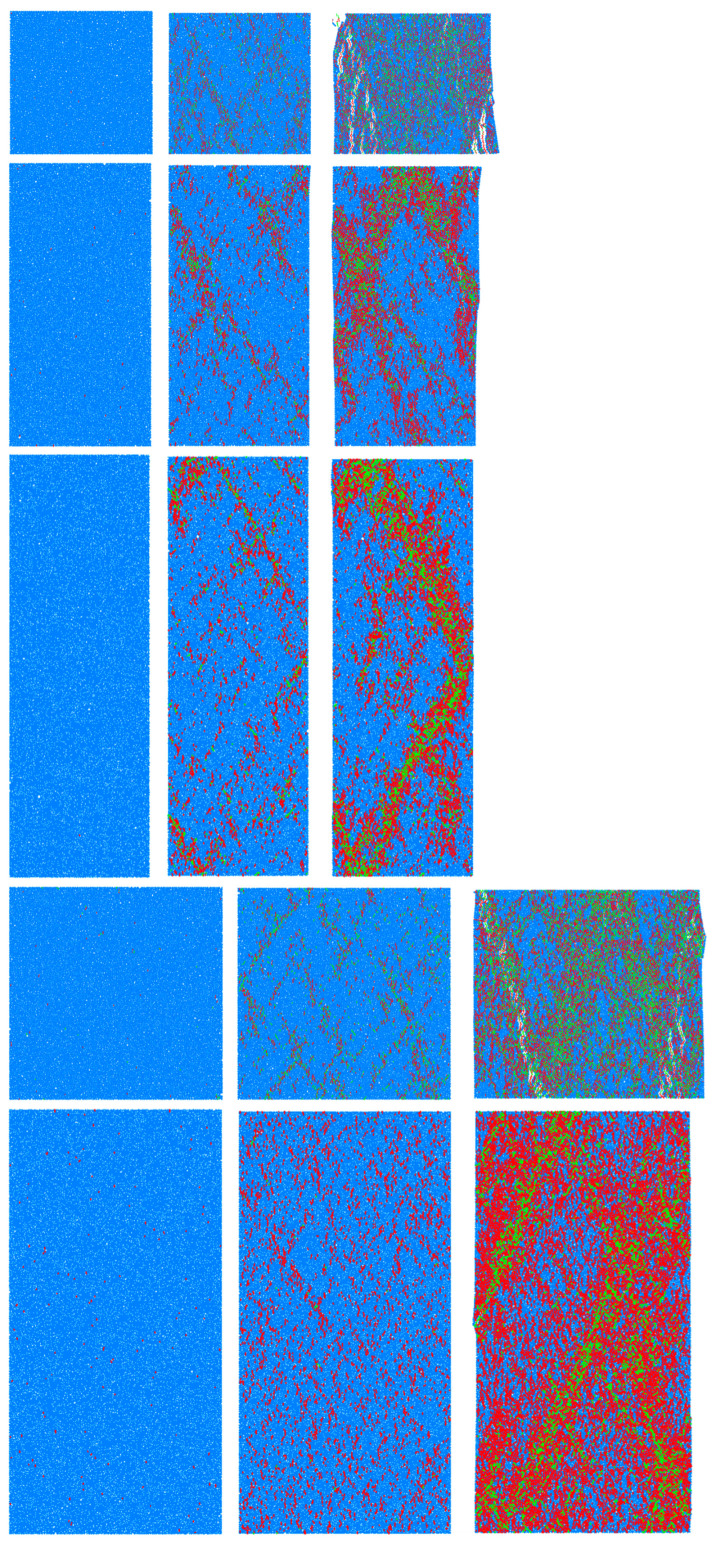
The crack evolution process in the model at 0.5 times the peak stress, the peak stress, and the post-peak residual stress of 0.5 times the peak stress.

In Figure 13, the sequence from left to right is the crack evolution process in the model at 0.5 times the peak stress, the peak stress, and the post-peak residual stress of 0.5 times the peak stress. Red represents tensile cracks, and green represents shear cracks. The result of numerical simulation is that at 0.5 times the peak stress, there are only a few tensile cracks in the model. At the peak stress, there are more tensile cracks and a small amount of shear cracks in the model. At the post-peak residual stress of 0.5 times the peak stress, the failure of different size models is quite different. The failure mode of the model with the size of 100 mm × 100 mm × 100 mm is mainly tensile. There are many tensile cracks parallel to the compression direction, which are extended and penetrated. The overall failure of the model is very serious. There are a large number of shear cracks concentrated on the top of the model in the model with a size of 100 mm × 100 mm × 200 mm, and failure of the top of the model is serious. There are few cracks at the bottom of the model, and most of them are tensile cracks. The overall integrity of the model after failure is more complete than that of the model with a size of 100 mm × 100 mm × 100 mm. There is an obvious oblique crack in the model with the size of 100 mm × 100 mm × 300 mm, which is mainly shear crack and penetrates from the top to the bottom of the specimen, and the shear failure characteristics are obvious. The overall integrity of the model after failure is more complete than that of the model with a size of 100 mm × 100 mm × 200 mm. There are a large number of tensile and shear cracks in the model with the size of 150 mm × 150 mm × 150 mm. The large-scale macroscopic penetrating cracks formed by the expansion of tensile cracks. Compared with the model with a size of 100 mm × 100 mm × 100 mm, the overall integrity of the model after failure is more complete. There is a large number of tensile cracks with uniform distribution in the model with the size of 150 mm × 150 mm × 300 mm, and shear cracks with concentrated distribution and form large-scale oblique cracks. Compared with the model with a size of 100 mm × 100 mm × 200 mm, the overall integrity of the model after failure is more complete.

### 4.3. Contact Force Chain

**Figure 14:**
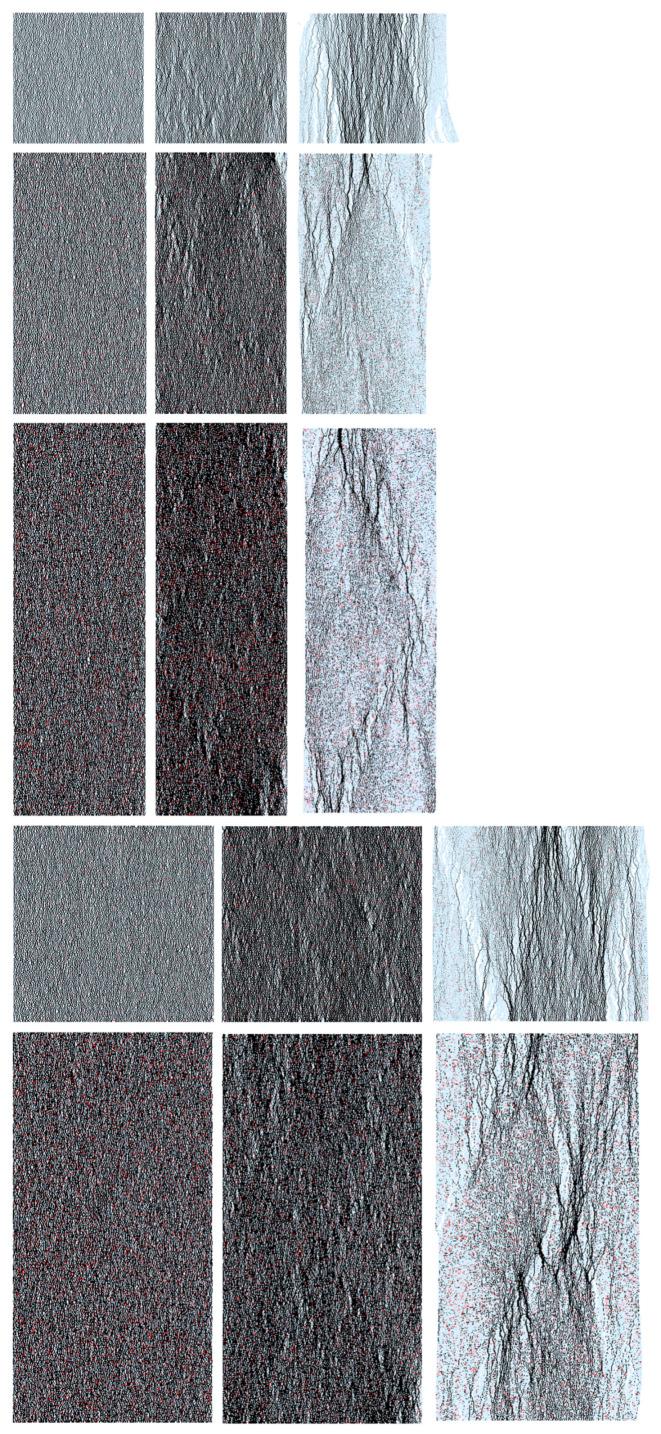
The contact force chain in the model at 0.5 times the peak stress, the peak stress, and the post-peak residual stress of 0.5 times the peak stress.

In Figure 14, the sequence from left to right is the contact force chain in the model at 0.5 times the peak stress, the peak stress, and the post-peak residual stress of 0.5 times the peak stress. The red force chain represents tensile stress, and the black force chain represents compressive stress. The darker the color, the higher the degree of stress concentration. At 0.5 times the peak stress, the distribution of the contact force chain in the model is relatively uniform, with a low degree of concentration. At peak stress, the distribution of the contact force chain in the model is relatively concentrated. At the post-peak residual stress of 0.5 times the peak stress, the concentration of the contact force chain is highest. The width of the model is the same, and as the aspect ratio increases, the concentration of the contact force chain becomes higher. As the degree of damage increases, the concentration of the contact force chain becomes higher.

## 5. Conclusions

In this paper, the influence of specimen size on the failure characteristics and mechanics of plastic concrete was investigated through uniaxial compression tests and numerical simulations. The results obtained from both the numerical simulations and indoor experiments were found to be consistent and in agreement with each other.

When the bottom size of the specimen is the same, with the increase in the height–width ratio (from 1 to 3), the peak stress of the specimen gradually decreases, but when the decrease is greatly reduced, the concentration of contact force chains in the model increases. The failure mode of the specimen changes from tensile failure to shear failure, and the distribution of cracks changes from multiple vertical cracks uniformly to a concentrated main oblique crack. The failure characteristics change from the overall failure to the serious failure of the near stressed end of the specimen, while the far stressed end is not a failure or a slight failure.

When the height–width ratio of the specimen is the same, with the increase in the overall size, the peak stress of the specimen decreases. When the number of cracks decreases, and the dense vertical cracks change into a small amount of concentrated oblique cracks, the integrity of the specimen and model is better.

The numerical simulation conducted using PFC2D software can provide a more detailed observation and analysis of the crack evolution and failure characteristics of plastic concrete in experiments, as well as the mechanical behavior during the experimental process. It can also reveal the deformation and failure process of specimens under compression loading, which can provide important references for further understanding the mechanical behavior of plastic concrete and support related engineering design and analysis. By comparing with experimental results, the accuracy and applicability of using PFC2D software for numerical simulation of plastic concrete have been verified.

## Figures and Tables

**Table 1:** Indicators of cement.

Item	Compressive Strength (MPa)		Flexural Strength (MPa)		Setting Time (h: min)		Water Consumption of Standard Consistency/%
	3 Days	28 Days	3 Days	28 Days	Initial Setting	Final Setting	
test value	24.8	48.3	5.8	9.1	1:55	3:55	27.5

**Table 2:** Indicators of bentonite.

Mud-Making Rate (m^2^/T)	Viscosity (op)	Water Loss (mL)	Sand Content (%)	Particle-Size (Mesh)	Water Content (%)	Colloid Value (%)	Blue Absorption (g/100 g)	Expansion Multiple
>16	≥15	<13	≤0.5	200	<10	<70	<39	>10

**Table 3:** The screening test results of sand.

**sieve aperture/mm**	9.5	4.75	2.36	1.18	0.60	0.30	0.15
**percentage of accumulated sieve residues (%)**	0	5.7	22.9	36.5	62.8	80.7	94.1

**Table 4:** The screening results of gravel.

**sieve aperture/mm**	63	31.5	26.5	19.0	16	13.2	9.5	4.75	2.36
**percentage of accumulated sieve residues (%)**				6.1	19.1	38.5	63.2	92.9	98.4

**Table 5:** Mix proportions.

Material Contents/kg·m^−3^				
Bentonite	Cement	Sand	Gravel	Water
95	155	795	795	285

**Table 6:** The peak stress of the specimens.

No.	Specimen Size/mm	Height–Width Ratio	Peak Stress/MPa
P4-59	100 × 100 × 100	1	10.61
P4-60	100 × 100 × 100	1	10.70
P4-54	100 × 100 × 200	2	6.34
P4-57	100 × 100 × 200	2	6.35
P4-56	100 × 100 × 300	3	5.59
P4-62	100 × 100 × 300	3	5.31
P4-63	150 × 150 × 300	2	6.21
P4-64	150 × 150 × 300	2	6.24
P1-11	150 × 150 × 150	1	7.26
P1-18	150 × 150 × 150	1	7.46

**Table 7:** Microscopic parameters.

Parameters	Value
Minimum particle diameter (mm)	0.25
Particle diameter ratio	4.0
Grain density (g/cm^3^)	2.72
Contact modulus of the particle (MPa)	9.85
Contact bond gap (mm)	0.05
Porosity	0.08
Parallel bond friction angle (°)	30.5
Parallel bond tensile strength (MPa)	0.82
Normal critical damping ratio	0.5
Parallel bond cohesive force (MPa)	3.27

## Data Availability

The original contributions presented in the study are included in the article, further inquiries can be directed to the corresponding author.
